# Case study on the use of intensive pediatric neurorehabilitation in the treatment of kernicterus

**DOI:** 10.1186/s40734-020-0084-z

**Published:** 2020-02-03

**Authors:** Jessie Mann, Dory A. Wallace, Stephanie DeLuca

**Affiliations:** 1 Translational Biology, Medicine & Health Program, Virginia Tech Carilion, Fralin Biomedical Research Institute, 2 Riverside Circle, Roanoke, VA 24016 USA; 2 Neuromotor Research Clinic, Fralin Biomedical Research Institute, Virginia Tech Carilion, 2 Riverside Circle, Roanoke, VA 24016 USA; 3 Virginia Tech School of Medicine, Virginia Tech Carilion, Fralin Biomedical Research Institute, 2 Riverside Circle, Roanoke, VA 24016 USA; 4 Virginia Tech School of Neuroscience, Virginia Tech Carilion, Fralin Biomedical Research Institute, 2 Riverside Circle, Roanoke, VA 24016 USA

**Keywords:** Intensive pediatric Neurorehabilitation, Kernicterus, Kernicterus Spectrum disorder

## Abstract

**Background:**

Kernicterus Spectrum Disorder (KSD) is the result of prolonged bilirubin toxicity resulting in widespread neurological injury. Once the bilirubin levels are normalized the encephalopathy becomes static, however the consequences of the injury can have life-long effects. The sequelae of KSD include motor impairments, auditory deficits, dental dysplasia, and potentially cognitive impairments. While KSD is a rare diagnosis, particularly in developed countries, there is evidence that there may be a global increase in incidence (Hansen, Semin Neonatol 7:103–9, 2002; Johnson, J Perinatol 29:S25–45, 2009; Kaplan etal. Neonatology 100:354–62, 2011; Maisels, Early Hum Dev 85:727–32, 2009; Olusanya etal., Arch Dis Child 99:1117–21, 2014; Steffensrud, Newborn Infant Nurs Rev 4:191–200, 2004). The literature on the treatment of various specific sequelae of KSD is varied, but in general specific therapeutic efforts to improve motor skills are not evidenced-based. The following is a case report on the use of Acquire therapy, an intensive neuromotor intervention, to ameliorate some of the motor-function deficits secondary to KSD.

**Case presentation:**

This case-report presents the results of two intensive therapeutic intervention sessions in one male child with KSD. Treatments occurred at 28 and 34 months. The child presented with fine and gross motor deficits as well as communication delays. Each session consisted of daily therapy for 4 h each weekday for 3 weeks. The child was assessed before and after treatment with 2 standardized measures, the Gross Motor Function Measure (GMFM) and The Bayley Scales of Infant and Toddler Development (Bayley).

**Conclusions:**

The GMFM at the 1st assessment was 34, 74at the 2nd assessment (after intervention 1), and 64 at the third assessment and 104 at the 4th assessment (after intervention 2). The Bayley at the 3rd assessment was 18, and 38 at the 4th assessment (after intervention 2).

## Background

Kernicterus, or kernicterus spectrum disorder (KSD) is the neurological sequelae of bilirubin toxicity [1]. Eighty-five percent of newborns are affected by unconjugated hyperbilirubinemia which results in jaundice. Treatment of acute bilirubin encephalopathy (ABE) typically involves exchange transfusion and phototherapy [2]. The diagnosis of kernicterus stems from serum bilirubin levels exceeding 20–25 mg per 100 ml [3, 4] for an extended period of time [4] causing damage to the developing brain [5]. This damage, caused by ongoing ABE is often widespread and neural degeneration is progressive and can lead to death without treatment [6]. If the ABE is identified and bilirubin levels are normalized the encephalopathy becomes static, but the previous damage to the developing brain can have lifelong implications (Ibid) causing various levels of developmental disability. The development of kernicterus is considered a “never event” by the medical community, nevertheless, it continues to occur in both developed and developing nations [7–12]. Kernicterus has an incidence between 0.4 and 2.7 cases per 100,000 births in the United States [5] and in the developing world it can account for 15% of neonatal deaths [6]. The clinical kernicteric sequelae of chronic ABE can include non-progressive: hearing loss, dental dysplasia, athetosis, choreoathetosis, paralysis of upward gaze, dystonia, spasticity, hypotonia, and, rarely, intellectual deficits [2, 4, 10, 13]. Motor impairments associated with Kernicterus often result from lesions of the subthalamic nucleus, globus pallidus, cerebellum and brain stem nuclei [2]. The presentation of motor impairments across children with Kernicterus are varied with regard to type and severity, but in children with moderate to severe motor impairment they often present with a mix of hyperkinetic movements that limit gross and fine motor skill acquisition.

For children with KSD, long term care often includes multi-disciplinary rehabilitation involving speech therapy, auditory interventions, as well as physical, occupational, and pharmacological therapy (Usman, Diala, Shapiro, Pichon, & Slusher, 2018). However, there is little to no documented efficacy for directly improving functioning in children with KSD. In other neuromotor etiologies, such as Cerebral Palsy and microcephaly, intensive bursts of neurorehabilitation have resulted in increased functional motor and cognitive skills [14–18], and we hypothesize that intensive neurorehabilitation could promote motor skill acquisition, decrease the presence of unwanted movements, and improve functionality in young children with Kernicterus.

## Case presentation

### Study design

This case study is an ABA design that involves two clinically implemented intensive treatments separated by 6 months in 1 male child. The child was 28 months of age for the first treatment and 34 months for the second. The collection and use of data was approved by the University’s Institutional Review Board, and parents provided consent for treatment and the use of all data gathered during treatment.

### Participant

The child has been diagnosed with dystonia, auditory neuropathy, and kernicterus. A CT scan performed at 8 days of age demonstrated mild cerebral edema and hyperdense cerebral venous sinuses, both of these issues were resolved at a follow up CT delivered 2 days later. He presented with limitations in gross and fine motor skills, as well as speech production. The child has choreoathetoid movements that interfered with targeted reach and grasp, as well as balance, during both pre-treatment assessments. He receives the current standard of care for pediatric rehabilitation which includes physical therapy 3 times a month for an hour, occupational therapy 2 times a month for an hour, and speech therapy 2 times a month for an hour. He began receiving standard of care at 3 months of age. Additionally, he is being treated with stem cell therapy twice a year outside of the U.S.

Parental goals for treatment included improvements in; sitting balance, ability to transition between supine and sitting, mobility via crawling, communication, and self-feeding. At the first assessment (28 months) the child was unable to sit independently, maintain head control, or self-feed. At the second treatment session the child was 34 months of age and could sit unassisted but needed supervision due to the instability associated with the interference of hyperkinetic movements that would destabilize him. He could self-feed, but could not drink without assistance and had recently gained a few dozen words. He could commando crawl to objects of interest, but could not pivot when sitting. Parental goals for session two included independent drinking, crawling, improved postural stability and sitting balance, improved ability to transition between positions, and improved communication.

### Treatment protocol

**Fig. 1:**
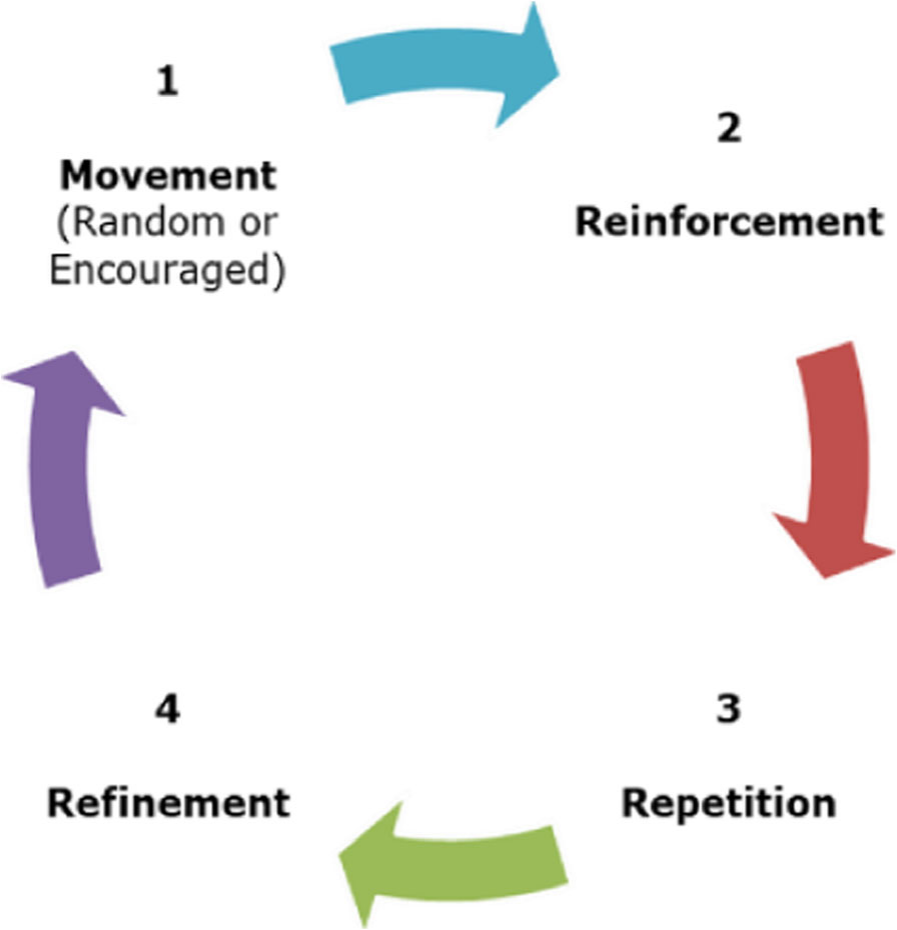
The MR3 Cycle

Treatment was delivered for 4 h a day for 2 consecutive work weeks, the family was traveling on the third week and returned for another week of treatment thereafter, for a total of 3 weeks of treatment in the first session. In the second session the child received 4 h of treatment a day for 15 days of consecutive weekday treatments. Treatment was based on the Acquire treatment protocol [18] which includes a high dosage of therapy, the use of shaping techniques and repetitive practice. Operant conditioning is used to guide treatment activities. Specifically, the Acquire MR3 cycle is an operant conditioning model using reinforcement, repetition, and refinement [18], see Fig. 1. Treatment involves reinforcing the production of starting functional movements, the repetition of these movements which are then progressively refined or shaped towards increasing proficiency incrementally. All treatment sessions also took place in a natural setting, and activities were play-based and goal-directed. Treatment focused on improving motor skills, such as postural control, independent sitting, and motor transitions, as well as control over unwanted movements or the inhibition of movements that interfered with the successful production of functional outcomes. The child was encouraged to ‘use his words’ or signs where appropriate to improve communication abilities.

### Assessments

#### Standardized assessment

The child was assessed before and after treatment for both treatment sessions using the Gross Motor Function Measure (GMFM) a standardized observational instrument. The assessment focuses on the areas of lying, sitting, crawling, standing, and walking/running/jumping. Because the child is not yet standing or walking our GMFM assessment focused on the first three areas of assessment. The GMFM has been validated for children with cerebral palsy between the ages of 5 mo. and 16 years [19, 20]. However, it is generally accepted that is sensitive to changes in children with other neuromotor impairments. For the second treatment session the Bayley Scale of Infant and Toddler Development was added, which assesses Cognitive, Expressive Language, Receptive Language, Fine motor and Gross motor developmental scales. The Bayley has been validated for use with children ages 1–42 months [21].

#### Qualitative assessment

Qualitative assessments were made through the consensus of the care team and parental observation and only those changes recorded in the treatment notes and confirmed by the family are here reported.

#### Goal-specific assessment

Treatment video analysis for goal specific changes was performed for sessions 1 & 2. Treatment video from the first and last weeks of each treatment sessions were analyzed to determine changes in the child’s ability to gain and maintain sitting balance, and the child’s gains in terms crawling and communication. Video segments were selected based on the task demand, such that for the goal of sitting balance, only those clips where the child was being asked to maintain a seated position were selected for possible analysis. These video windows were then randomly sampled for analysis. Video windows of 5 min in length were generated for the assessment of sitting duration, and the longest period of uninterrupted sitting balance was measured and compared between the first and last weeks of treatment. Moreover the average length of independent sitting across analysis windows was compared between first and last weeks. For sitting frequency, video windows of 20 min were generated and used to measure the ratio of time spent in independent sitting balance per 20 min. Sitting balance was defined as sitting without the assistance of a supportive device or individual. The child was considered to be in independent sitting balance if he was propping himself with the use of his hand on the floor, bench, or his legs. The treatment team could provide blocking to the knees, or thighs, but could not provide support to assist in sitting. Communication was assessed by counting the number of clear signs or words produced in a 5 min window of treatment video. Crawling was assessed by selecting video from the first and last weeks of treatment where the child was encouraged to independently move toward an object of interest and measuring the distance covered. In the analysis of video from the first week the child had a starting value of zero, he was unable to move independently in a crawling pose. Because the starting value was zero a change score couldn’t be calculated but the analysis of video from the last week of treatment revealed a functional gain of 3 feet of independent crawling, as reported on in Fig. 4.

## Conclusions

**Fig. 2:**
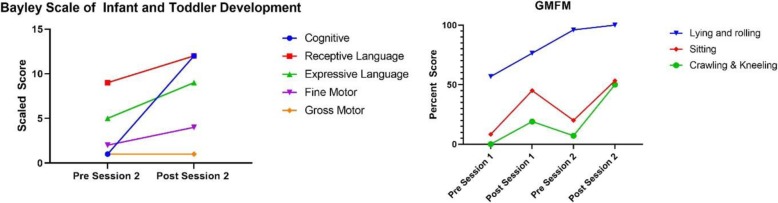
Raw Score Change Standardized Assessments

**Fig. 3:**
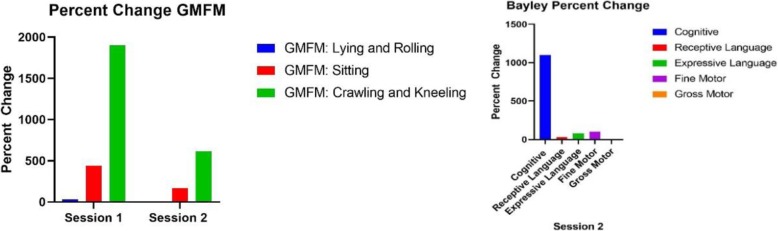
Percent Change on Standardized Assessments

**Fig. 4:**
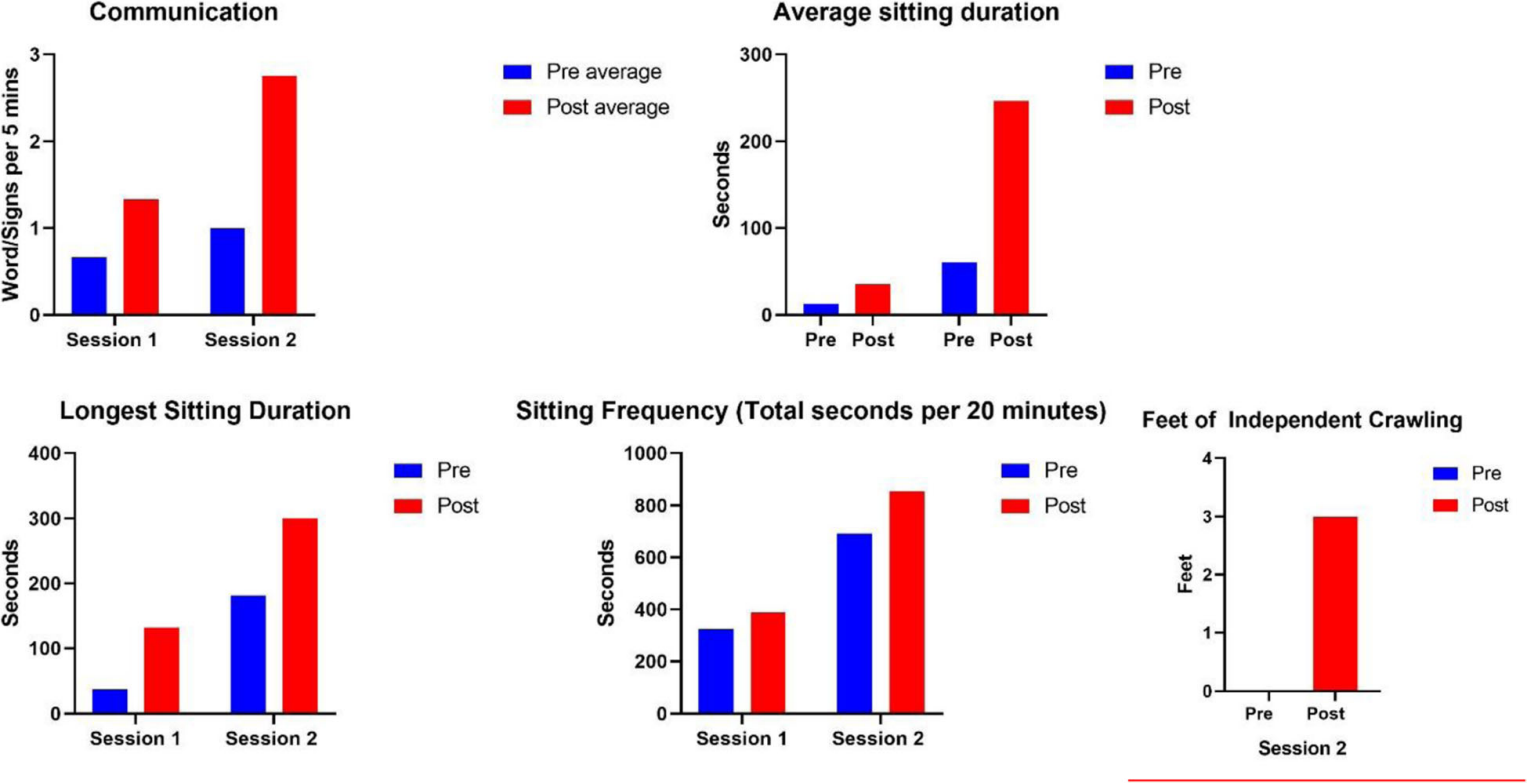
Goal Specific Measurement Changes

**Fig. 5:**
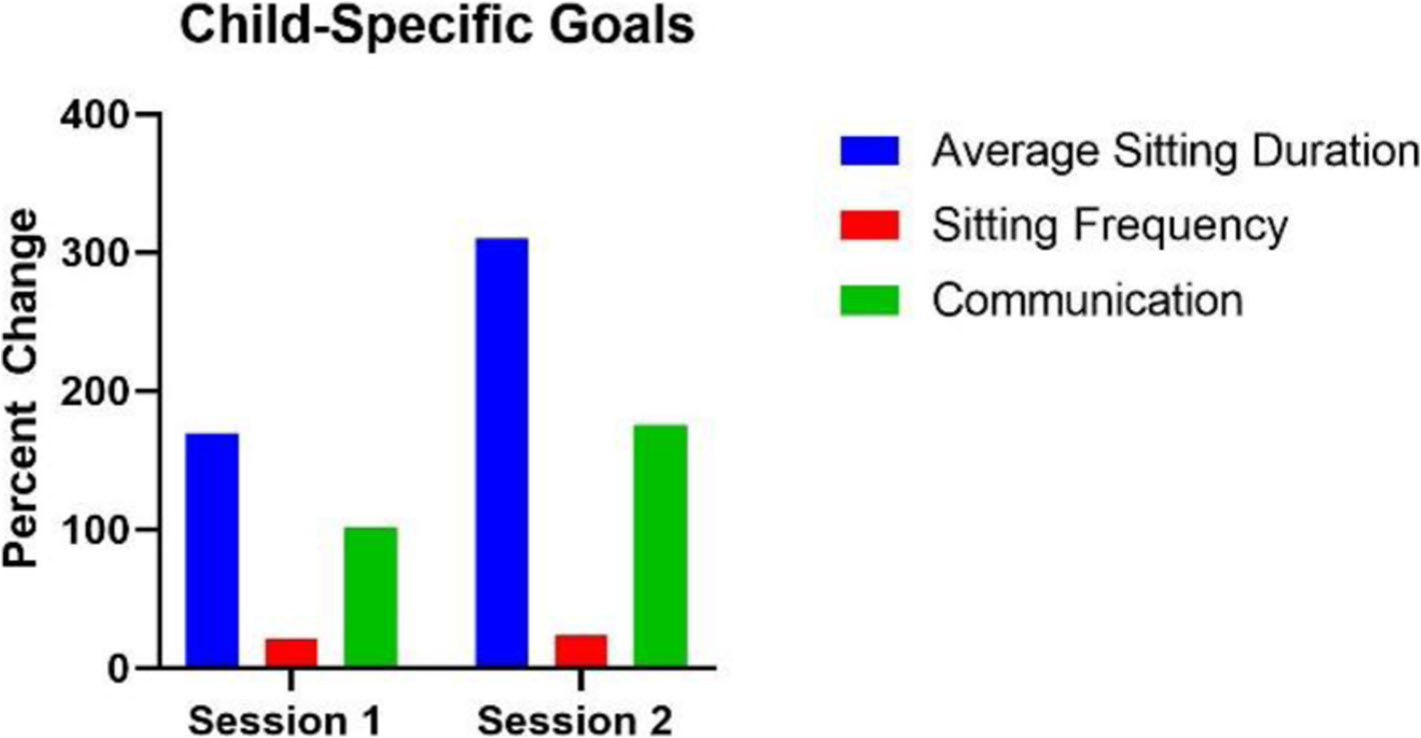
Percent Change on Goal Specific Measurements

The child’s pre-treatment and post-treatment GMFM and Bayley scores are reported in Table 1, and Figs. 2 & 3 show percent change on standardized assessments. Qualitative assessments are reported in Table 2. Goal specific changes are reported in Figs. 4 & 5.

**Table 1:** GMFM and Bayley Scores

GMFM (Raw Score)	Lying & Rolling	Sitting	Crawling & Kneeling		
Session 1 Pre	29	5	0		
Session 1 Post	39	27	8		
Session 2 Pre	49	12	3		
Session 2 Post	51	32	21		
Bayley Scaled Score	Cognitive	Receptive Language	Expressive Language	Fine Motor	Gross Motor
Session 2 Pre	1	9	5	2	1
Session 2 Post	12	12	9	4	1

**Table 2:** Qualitative Assessment of Functional Gains

Session 1	Session 2
Gained ability to cross midline during play- Noted on week 1	Improved targeted reaching Noted on week 4
Increased tolerance for and control in quadruped position – Noted on week 1	Improved grasp – Noted on week 1
Improved targeted reaching- Noted on week 2	Improved bimanual engagement- Noted on week 1 & 3
Improved grasp – Noted on week 2	Gained in ability to engage in protective extensions- Noted on week 1
Gained ability to engage in bilateral hand play at midline- Noted on week 2	Gained the ability to shift to a toy and come back to sitting- Noted week 3
Gained ability to inhibit unproductive movements- Noted on week 2	Gained the ability to pivot to toys – Noted on weeks 3 &4
Gained ability to self-feed finger foods- Noted on week 2	Gained the ability to operate a tricycle – Noted on week 4
Increased head control – Noted on week 2	Gained the ability to drink with minimal assistance- Noted on week 3
Increased use of left hand for stabilization for sitting- Noted at 40–50% of the time on week 2

## Discussion

The functional gains made by this child, on both standardized assessments and goal-specific assessments, suggest that further research on the use of intensive neuromotor rehabilitation for children with Kernicterus is warranted. This case report contributes to the growing body of literature that suggests that intensive bursts of therapeutic interventions are appropriate for use with various neuromotor disorders of diverse etiologies. In all goal-specific domains the child maintained skills between sessions and differential responsiveness to certain domains of function by treatment session provides data suggestive of the importance of repeated bursts of intensive interventions at different developmental periods. Moreover, the child’s improvements in sitting balance are reflective of a functional decrease in unwanted movements. These results support further, more highly powered and controlled research into the use of intensive therapeutic bursts of neuromotor intervention for the treatment of the sequelae of KSD. Specifically, the authors propose that this intervention improved the child’s ability to minimize these hyperkinetic movements, as demonstrated by the gains in sitting balance, which had previously been disrupted by these hyperkinetic movements. This proposal is being explored further in an ongoing follow-up study. The authors would also like to suggest that the reportingof qualitative, standardized, and goal-specific assessment as done in this case report, greatly improves the quality and specificity of such a case report, and that, moreover, the inclusion of a change score improves the therapeutic interpretation of the data, allowing comparisons of interventional effect by goal, by functional domain, and by treatment session. The child’s family reported that his marked improvement in communication, sitting balance, and movement (in terms of gaining the ability to crawl and use a tricycle after the second treatment session) dramatically altered the child’s ability to interact with others and the environment around him, allowing him much greater participation in the home and social environment and increasing his ability to self-direct his activities.

### Limitations

While a single care report is insufficient for the justification of a treatment intervention or the redistribution of therapeutic hours, this preliminary data is suggestive that the distribution of therapeutic hours and repeated bursts of intervention might be an important consideration for the pediatric rehabilitation community. However, this single case report lacks a control or a means to account for an ongoing baseline rate of developmental change. The follow-up study, currently underway, is including a measurement of subjects’ baseline rate of change, for the 6 weeks prior to and after the treatment intervention so that the rate of change in usual and customary care can be compared to the rate of change during the intensive intervention. Moreover, more specific attention to the assessment of hyperkinetic movements has been implemented so as to explore further the authors’ suggestion that the reported functional gains are associated with a gain in control over these unwanted movements.

## Data Availability

The datasets supporting the conclusions of this article are included within the article and its additional files.
